# Fluorescence Imaging of Epidermal Growth Factor Receptor Tyrosine Kinase Inhibitor Resistance in Non-Small Cell Lung Cancer

**DOI:** 10.3390/cancers14030686

**Published:** 2022-01-28

**Authors:** Marisa L. Martin-Fernandez

**Affiliations:** Central Laser Facility, Science & Technology Facilities Council, Rutherford Appleton Laboratory, Didcot OX11 0FA, UK; martin-fernandez@stfc.ac.uk

**Keywords:** EGFR, tyrosine kinase inhibitors, fluorescence microscopy, super-resolution, autophagy, endocytosis, apoptosis, protein aggregation, protein conformation

## Abstract

**Simple Summary:**

Lung cancer is the leading cause of cancer-related deaths, with a low (<21%) 5-year survival rate. Lung cancer is often driven by the misfunction of molecules on the surface of cells of the epithelium, which orchestrate mechanisms by which these cells grow and proliferate. Beyond common non-specific treatments, such as chemotherapy or radiotherapy, among molecular-specific treatments, a number of small-molecule drugs that block cancer-driven molecular activity have been developed. These drugs initially have significant success in a subset of patients, but these patients systematically develop resistance within approximately one year of therapy. Substantial efforts towards understanding the mechanisms of resistance have focused on the genomics of cancer progression, the response of cells to the drugs, and the cellular changes that allow resistance to develop. Fluorescence microscopy of many flavours has significantly contributed to the last two areas, and is the subject of this review.

**Abstract:**

Non-small cell lung cancer (NSCLC) is a complex disease often driven by activating mutations or amplification of the epidermal growth factor receptor (EGFR) gene, which expresses a transmembrane receptor tyrosine kinase. Targeted anti-EGFR treatments include small-molecule tyrosine kinase inhibitors (TKIs), among which gefitinib and erlotinib are the best studied, and their function more often imaged. TKIs block EGFR activation, inducing apoptosis in cancer cells addicted to EGFR signals. It is not understood why TKIs do not work in tumours driven by EGFR overexpression but do so in tumours bearing classical activating EGFR mutations, although the latter develop resistance in about one year. Fluorescence imaging played a crucial part in research efforts to understand pro-survival mechanisms, including the dysregulation of autophagy and endocytosis, by which cells overcome the intendedly lethal TKI-induced EGFR signalling block. At their core, pro-survival mechanisms are facilitated by TKI-induced changes in the function and conformation of EGFR and its interactors. This review brings together some of the main advances from fluorescence imaging in investigating TKI function and places them in the broader context of the TKI resistance field, highlighting some paradoxes and suggesting some areas where super-resolution and other emerging methods could make a further contribution.

## 1. A Brief Outline of Non-Small Cell Lung Cancer 

Lung cancers are classified in two main histological groups: small-cell lung cancer (SCLC) and non-small cell lung cancer (NSCLC) [1]. SCLC comprises ~15–20% of all primary lung tumours and is often caused by smoking [2]. NSCLC frequently arises among non-smokers and can be sub-divided into adenocarcinoma, squamous cell carcinoma, the most prevalent, large cell carcinoma, and bronchial carcinoid tumour (reviewed in [3]). Like all tumours, dysregulated cell division is driven in NSCLC by genetic alterations, the accumulation of which eventually enables tumour cells to acquire limitless replicative potential (reviewed in [4]). Gene sequencing technologies have allowed the identification of driver oncogenic gene alterations in the *EGFR* gene itself (reviewed in [5]), and/or of genes expressing oncogenic proteins within EGFR’s downstream signalling pathways, especially those that regulate cell survival and proliferation, on which tumour initiation and growth critically depend [6] (examples in Box 1). Mutations in genes downstream of *EGFR* decouple cell growth and proliferation from EGFR signalling, hence anti-EGFR drugs become ineffective.

Box 1 **Some Common Oncogenes in NSCLC.**

***EGFR***
EGFR is one of the four members of the human epidermal growth factor (HER) family transmembrane receptors (HER1/EGFR, HER2, HER3, and HER4). The prevalence of *EGFR* oncogene mutations is 50% among Asian patients with lung adenocarcinoma and 15% among Western patients [7]. Exon 19 deletions or L858R point mutations in exon 21 account for 90% of the activating mutations in the tyrosine kinase domain of EGFR, resulting in constitutive activation of EGFR without growth factor-induced stimulation, thus promoting cell proliferation [5].
***KRAS***
*KRAS* is the predominantly mutated RAS isoform (85%) and also the most frequent oncogene in NSCLC [8]. *KRAS* fosters tumour growth via several mechanisms, including by upregulating rate-limiting enzymes involved in amino acid, fatty acid, or nucleotide biosynthesis, and by stimulating scavenging pathways, such as macropinocytosis and autophagy [9,10], which, in turn provide building blocks for the anabolic routes, also maintaining the energy levels and the cell’s redox potential [11].
***BRAF***
*BRAF* is a proto-oncogene encoding a serine-threonine protein kinase acting downstream of the RAS/RAF/ERK signalling pathway. BRAF carries signals from membrane receptors (such as EGFR) to the nucleus of the cell to regulate DNA transcription [12]. BRAF is an oncogene located on chromosome 7 involved in several cell functions, including growth, proliferation, survival, and differentiation. Immunotherapy is beginning to show promise as an active therapy in *BRAF*-mutated NSCLC [13].
***ALK***
The *ALK* gene encodes the ALK tyrosine kinase receptor and is associated with many types of cancers, including NSCLC [14]. There are three types of *ALK* mutations: rearrangement (ALK-R), amplification (ALK-A), and point mutation. ALK gene rearrangement is a driving mutation underlying the development of NSCLC [15], which appears to be more common in younger patients and never or light smokers diagnosed with adenocarcinoma. ALK can phosphorylate STAT3 and PI3K independently of ERK to antagonise apoptosis and promote cell survival [16].
***TP53***
The *TP53* gene encodes a DNA damage check point p53 protein, which is at the heart of the cellular decision to proliferate or activate programmed cell death. It regulates the transcription of ~500 genes [17], including cell cycle regulatory genes and transcription factors, and DNA repair genes [18]. Over 50% of human cancers carry loss of function mutations in *TP53*, with the mutant form acting as a dominant-negative inhibitor towards the wild-type moiety. When chromosomal abnormalities or environment stresses become overwhelming, p53 can arrest cell-cycle progression and induce apoptosis. *TP53* alterations carry a worse prognosis in NSCLC [19].
***MYC***
A family of three human proto-oncogenes (*c*-*MYC*, *l*-*MYC*, and *n*-*MYC*) code for transcription factors [20]. In normal cells, depending on nucleotide pools’ levels, growth signals, glucose, or oxygenation, elevated MYC expression can cause apoptosis. Transformed cells can, however, adapt to constitutively elevated levels of MYC expression, resist its apoptotic effects, and only respond to MYC pro-proliferative signals either via loss of growth suppression surveillance mechanisms (e.g., *TP53* mutation) and/or by gain of pro-survival signals. *MYC* is a metastasis gene for NSCLC [21].

Surgery, radiation, and chemotherapy remain among the first-line treatments for NSCLC [22]. More targeted therapies include immune check-point inhibitors, engineered cytotoxic chimeric antigen receptor-immune T cells, oncolytic viruses, anti-tumour vaccines, and small-molecule inhibitors against oncogenes driving NSCLC tumours (reviewed in [23]). Of interest here is the sub-class of quinazoline-derived small-molecule EGFR-selective tyrosine kinase inhibitors (TKIs) that target EGFR, and specifically first-generation gefitinib and erlotinib, because these two TKIs are still commonly employed as first-line therapies (reviewed in [24]) and have also been extensively investigated via fluorescence microscopy methods (Box 2). 

**Figure 1:**
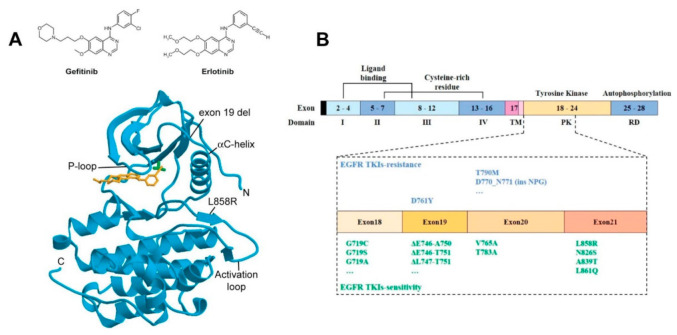
Gefitinib and erlotinib binding to EGFR’s kinase domain. (**A**) Top: Structures of gefinitib and erlotinib; bottom: Schematic representation of the wild-type EGFR tyrosine kinase domain (cyan) bound to erlotinib (orange) (PDB entry 1M17). The threonine 790 side chain is shown in green (top right of the bound TKI). EGFR numbering includes the 24 residue signal sequence [35]. Conserved structural features essential to the activation of the kinase domain, the phosphate-binding loop (P-loop), the αC-helix, and the activation loop are shown. Sites of common NSCLC TKI-sensitive mutations (exon 19 deletion and L858R substitution) are also shown. Reproduced from [35]. (**B**) Schematic representation of the domains of EGFR and the corresponding exons. Specific NSCLC-related mutations in the kinase domain of EGFR (exons 18–24) that are associated with sensitivity or resistance to EGFR-TKIs are denoted [36]. Reproduced from [36].

EGFR signalling is at the heart of cell growth and proliferation. This makes EGFR mutations highly susceptible to be exploited by cancer cells to alter their physiology and achieve immortalisation (reviewed in [5]). Key oncogenetic *EGFR* alterations upon which NSCLC tumours become addicted to EGFR signals include somatic mutations in the *EGFR* gene clustered around the periphery of the catalytic adenosine triphosphate (ATP)-binding cleft in EGFR’s kinase domain (Figure 1A). The two most common are a point substitution in exon 21 (L858R), which, for example, accounts for 90% of all NSCLC activating oncogenic EGFR mutations in the NSCLC Caucasian patient subset, and an in-frame deletion in exon 19 (e.g., DL746-P750) [25] (Figure 1B). Lower-frequency mutations include point mutations in exon 18 (G719X, G719S, G719A) and exon 20 (V765A and T783A) (reviewed in [26]). Different mutations can display different sensitivities to TKI inhibition of autophosphorylation and downstream signals (see, for example, [27]).

The first tumour-suppressing responses to TKI therapy were observed for gefitinib and erlotinib almost 20 years ago (see, for example, [28,29,30]). Orally administered, these TKIs reversibly outcompete the binding of ATP to the phosphate-binding loop in the kinase domain of EGFR, thus suppressing its tyrosine kinase activity (reviewed in [31]) (Figure 1A). A striking response was found in a subset of ~10–40% of patients who harboured NSCLC tumours driven by somatic activating mutations in the first 4 exons of the tyrosine kinase domain of the *EGFR* gene [32,33] (Figure 1B). This success led to the approval of gefitinib and erlotinib for the treatment of NSCLC patients bearing such mutations [34].

Another common driver of NSCLC found in 50–90% of cases is an increase in the *EGFR* copy number, which often results in the overexpression of wild-type EGFR (wtEGFR) (reviewed in [37]). Among these tumours, approximately 80% were found to be de novo resistant to gefitinib and erlotinib despite TKIs potently blocking the kinase activity and autophosphorylation of wtEGFR [38]. The intrinsic resistance of wtEGFR-expressing tumours to TKIs is recapitulated by many other solid tumour types (reviewed in [39]). This is so even in the absence of mutations in effectors downstream of EGFR that decouple growth and survival pathways from EGFR signalling [15] (Box 1). The reasons for this are not well understood.

Box 2 **Exemplar Labelling Methods and Imaging Techniques to Ascertain Tki Function and Resistance in Cells.**

APOPTOSIS: Associated plasma membrane structural changes include translocation of the anionic phosphatidyl serine (PS) from the inner to the outer leaflet of the lipid bilayer where it can bind Annexin V, a Ca^2+^-dependent phospholipid-binding protein with high affinity for PS. By labelling Annexin V with fluorescent dyes (e.g., FITC [40]), one can image, for example, via wide-field or confocal microscopy, and/or flow cytometry, Annexin V-positive cells to determine the rate of apoptosis [41]. Fluorescent Annexin V conjugates provide a quick and reliable detection method of the early stages of apoptosis (reviewed in [42]). Apoptosis in cells can also be detected, for example, by imaging fluorescence conjugates of Bax as it translocates from the cytosol to the outer mitochondrial membrane, and/or cytochrome C as it is released from the mitochondria into the cytosol [43].

ENDOCYTOSIS: Immunostaining against endosomal protein markers (reviewed in [44]) includes against early endosome proteins (Syntaxin 6 and Rab5 [45], and EEA1 [46]), recycling endosome markers (e.g., Rab25 [47]), and late endosome/lysosomal markers (Rab7 [48], LAMP1 and LAMP2 [49], cathepsin D, and LIMPII [50]). Primary or secondary antibodies can be conjugated with dyes of different colours (e.g., Alexa 488, Alexa 594, or Alexa 647). Typical endosomes (~100 nm) are smaller than optical resolution (~250 nm), hence endosomes look like puncta under a wide-field or confocal fluorescence microscope. To image EGFR endocytic traffic, one can, for example, label an EGFR cognate ligand (e.g., EGF) with organic dyes, both visible and infrared [51], or clone EGFR with tags, such as Halo [52] and SNAP [53], which are subsequently labelled with Alexa or Cyanine dyes. In live cells, one can use fluorescent protein (FP) fusions of the endosomal markers and/or of other proteins (e.g., clathrin [54]). To image in the nucleus, a popular method is fluorescence in situ hybridisation (FISH) assays [55].

AUTOPHAGY: FP constructs of the 17 kDa soluble microtubule-associated protein 1A/1B-Light Chain 3 (LC3) [56] are commonly used (e.g., eGFP-LC3, mCherry-LC3, or RFP-LC3). During autophagy, the cytoplasmic form of LC3 (LC3-I) becomes covalently ligated to phosphatidyl ethanolamine (PE). The appearance of fluorescent puncta of the lipidated LC3-II form allow determination via wide-field or confocal fluorescence microscopy of the number of autophagosomes (dia. 500–900 nm [57]), where LC3-II is recruited to [58]. Serum depletion and the autophagic inhibitor 3-methyladenine (chloroquine) are often used as positive controls [59]. Colocalisation of red and green probes (e.g., RFP-LC3 and LysoSensor Green) allows for the morphological observation and quantification of autophagosome maturation and fusion with the lysosome [60]. pH-responsive FPs (and organic dyes) allow the evaluation of intracellular pH and interrogation of specific subcellular compartments [61].

SINGLE PARTICLE TRACKING (SPT): A direct probe of fluorescent particle movement in live cells (reviewed in [62]). In two colours, SPT can report molecular association and dissociation events in real time from which kinetic and dynamic interaction parameters can be determined (e.g., [63,64]). At the plasma membrane, SPT exploits total internal reflection fluorescence (TIRF) illumination to improve contrast (reviewed in [65]). Suitable organic dyes and other probes have to be selected to ensure specific interactions with the proteins of interest and to minimise non-specific staining of the (typically) glass surface where the TIRF evanescent wave illuminating the adjacent basolateral cell surface is concentrated [66,67]. SPT can also be used to track particles in endosomes and at the nucleus using probes, such as adaptamers and FPs, and/or bright organic dyes, such as Atto 647N (e.g., [68,69]).

NEAR-FIELD SCANNING OPTICAL MICROSCOPY (NSOM) [70]: The resolution of NSOM is defined by the size of the point light source used (typically 50–100 nm). NSOM breaks the far-field optical resolution limit (~250 nm) by exploiting the properties of evanescent waves in close vicinity (i.e., ~nanometres) of the aperture defining the size of the point light source, which must therefore be brought within nanometres of the surface to collect the near-field optical signal. The point source is scanned over the surface, without touching it. The distance between the point light source and the sample surface is usually controlled through a feedback mechanism that is unrelated to the NSOM signal (e.g., as in AFM) [71]).

STOCHASTIC OPTICAL RECONSTRUCTION MICROSCOPY (STORM) [72]: A single-molecule localisation microscopy (SMLM) method with a resolution of ~20 nm. It reports on the number of proteins that form nanoclusters and on the size of the clusters (example shown in Figure 6). STORM is compatible with many commonly used organic dyes, which can be converted to an off state using specific excitation parameters combined with oxygen-scavenging imaging buffers. Fluorophores for STORM should be bright, have a high rate of photo-switching, and exhibit minimal photo-bleaching in thiol-containing buffers. Normally used to analyse clusters in chemically fixed cells, sub-12 nm resolution is possible in cryo-vitrified samples using solid immersion lenses [73].

FLUORESCENCE RESONANCE ENERGY TRANSFER (FRET): A spectroscopic ruler useful for measuring intra-molecular and inter-molecular separations in the range ~2–8 nm [74]. It is based on the transfer of excitation energy between two fluorescent molecules through non-radiative dipole–dipole coupling [75,76]. The rate of energy transfer, from which the separation between donor and acceptor molecules can be measured, is determined chiefly from the overlap between the emission spectra of the donor and the excitation spectra of the acceptor. FRET can be combined with SPT [77,78,79] (Figure 6E), fluorescence lifetime imaging (FLIM) [80], and fluorescence polarisation [81]. The combination can be used to detect dimers and oligomers, and/or to determine separations between two planes, as a proxy for molecular orientation at the plasma membrane [82].

FLUOROPHORE LOCALISATION IMAGING WITH PHOTOBLEACHING (FLImP) [83,84]: Based on SMLM, the position of a cluster of fluorescent molecules changes upon each individual photobleaching event. The shift in the position of the cluster can be analysed to report on the lateral separations between the molecules in the cluster. FLImP can measure separations between identical fluorophores in the 0–60 nm range, and can achieve sub-5 nm resolution [85]. Combined with atomic molecular dynamics (MD) simulations, it can report on the dimer and oligomer structure [85,86].

## 2. EGFR’s Role in the Development of NSCLC Tumours 

### 2.1. EGFR Structure and Signalling Pathways 

**Figure 2:**
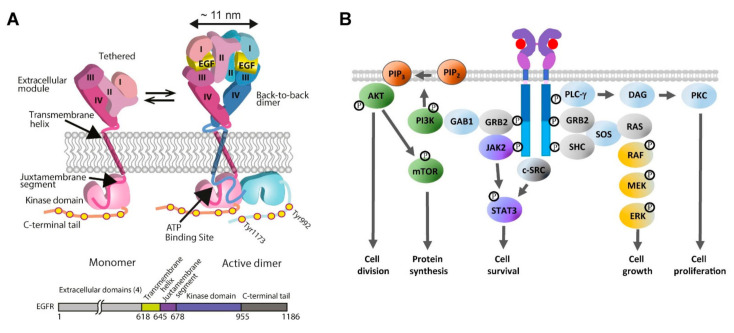
(**A**) Cartoon of the EGF-induced receptor dimerisation process and an EGFR sequence diagram. Left: A tethered single-pass EGFR monomer [93]. Right: The EGFR monomer binds EGF to form an extended back-to-back ectodomain dimer [94,95], structurally coupled via an N-crossing dimer of two transmembrane alpha-helices [99] to an asymmetric tyrosine kinase dimer [96], in which the activator kinase (pink) allosterically activates a receiver kinase (blue), which phosphorylates the C-terminal domain of the donor kinase [96,99]. Reproduced from [85]. (**B**) Growth factor-dependent EGFR signalling pathways. EGFR activates the RAS/extracellular signal-regulated kinase (ERK) pathway for cell growth, and the JAK/signal transducer and activator of transcription 3 (STAT3) signalling cascade for cell survival. Activation of the PI3K/AKT/mammalian target of rapamycin (mTOR) signalling pathway leads to cell division via AKT phosphorylation and protein synthesis via mTOR phosphorylation. EGFR activates Phospholipase C gamma (PLCγ), which in turn activates the PKC signalling pathway, leading to cell proliferation [100].

EGFR is the founding member of the family of four human receptor tyrosine kinases (HER1–4) (reviewed in [87]). Additionally termed ErbB1 because of its close similarity with the avian viral v-erb-B oncogene protein [88], EGFR was cloned and sequenced in the early 1980s [89], and is ubiquitously expressed in epithelial, mesenchymal, and neuronal cells (reviewed in [90]). Structurally, the EGFR consists of a growth factor-binding ectodomain made out of four subdomains, a single-pass transmembrane alpha helix, an inner juxtamembrane segment, a kinase domain locus of EGFR’s intrinsic protein tyrosine kinase activity, and a long unstructured C-terminal domain (reviewed in [91]) (Figure 2A). EGFR binds seven cognate growth factors, namely epidermal growth factor (EGF), transforming growth factor alpha, betacellulin, heparin-binding EGF-like growth factor, epiregulin, and epigen (reviewed in [92]). Growth factor binding induces a conformational change in EGFR’s ectodomain [93] that exposes a loop required for ectodomain dimerization [94,95] (Figure 2A). This leads to allosteric changes across the plasma membrane, chiefly the formation of a catalytically active asymmetric kinase domain dimer [96], via which EGFR becomes phosphorylated (p-EGFR) in five key C-terminal tyrosine phosphorylation sites (Tyr992, Tyr1045, Tyr1068, Tyr1086, and Tyr1173) [97,98]. Activating *EGFR* mutations and EGFR overexpression elicits growth factor-independent constitutive receptor dimerisation and/or oligomerisation, thereby activating the catalytic activity of the receptor without the need for the growth factor stimulus [86,99]. This allows EGFR to trigger downstream signalling pathways in a growth factor-independent dysregulated fashion, ultimately eliciting uncontrolled cell division and tumour proliferation [5,38]. 

Reviewed in [100], and summarised in Figure 2B, EGFR recruits via its C-terminal pY992 the Src Homology 2 (SH2) domain of PLC-γ, which hydrolyses PIP_2_, releasing diacylglycerol (DAG) and inositol 1,4,5-triphosphate (IP_3_), and leading to the activation of PKC and cell proliferation. EGFR can recruit via pY1068, pY1148, and pY1173 the SH2/SH3 adaptors GRB2 and SHC, which bind via their SH3 domains the protein scaffolds SOS and GAB1 to initiate well-defined tyrosine/serine/threonine phosphorylation cascades [101]. One is the RAS-RAF-MEK-ERK1/2 signalling pathway, which leads to ERK activation and translocation of ERK from the cytoplasm to the nucleus, where it upregulates genes that promote cell growth (reviewed in [102]). GRB2 also recruits via GAB1 the lipid kinase PI3K [103]. PI3K catalyses PIP_2_ into PIP_3_, which recruits AKT, leading to the activation of the PI3K-AKT-mTOR signalling pathway. Phosphorylation of AKT leads to the inhibition of antagonists of Cyclin D1 and cell division (reviewed in [104]). AKT-mediated phosphorylation of mTOR upregulates the cell’s anabolic metabolism (reviewed in [105]). Phosphorylated EGFR also activates the JAK2/STAT3 signalling axis to upregulate the transcription of a variety of proteins involved in the survival of cancer cells (reviewed in [106]). EGFR also interacts with c-SRC, a crucial non-receptor tyrosine kinase and an oncogenic partner in EGFR-driven NSCLC [107]. Among many other pro-survival functions [108], c-SRC synergises with EGFR to activate STAT3 in a JAK-independent manner [109].

### 2.2. TKI Treatments Induce Apoptosis via the Mitochondrial Intrinsic Pathway

**Figure 3:**
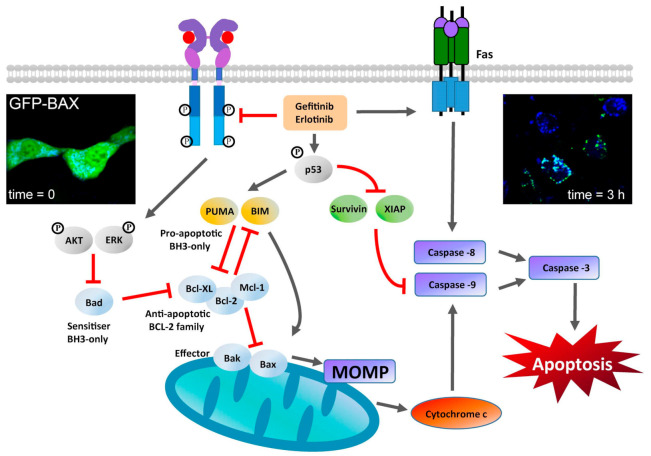
Stimulation of the mitochondrial-dependent intrinsic apoptosis pathway by gefitinib and erlotinib. This pathway is marked by a key event—mitochondrial outer membrane permeabilization (MOMP)—which results in the release of cytochrome c from the mitochondrial intermembrane space. MOMP can be triggered by the activation of BH3-only proteins of the BCL-2 family [112] following their post-translational modification (e.g., phosphorylation) [121]. Activated BH3-only proteins generally stimulate MOMP by inducing the oligomerization of BCL2-associated X protein (BAX) and/or BCL2 antagonist or killer (BAK) in the outer mitochondrial membrane, thereby forming supramolecular channels that mediate the liberation of cytochrome c [118]. At the cytosol, cytochrome c triggers the assembly of a caspase-activating complex between caspase 9 and apoptotic protease-activating factor 1 [122]. TKI inhibition can stimulate the transactivation of genes encoding pro-apoptotic proteins (such as the BH3-only protein p53-upregulated modulator of apoptosis (PUMA)). Gefitinib and erlotininib can also activate the so-called death receptor FAS, leading to activation of caspase 8 [111,112]. Caspase 8 proteolytically activates downstream effector caspases or truncates the BH3-only protein BID (BH3-interacting domain death agonist), which co-activates the intrinsic pathway of apoptosis by translocating to mitochondria. Caspase-8 interacts with caspase-9 to activate the executioner caspase-3, which coordinates the destruction of cellular structures, such as DNA fragmentation or degradation of cytoskeletal proteins [123]. Fluorescence image inserts: left: Live cell image of exogenous GFP-BAX expressed in D407 cells (immortalized human retinal pigment epithelial cells); right: same area imaged after inducing apoptosis using 1 μM staurosporine prepared in DMSO. Images were taking using a spinning disk confocal microscope, which is ideal for fast 3D imaging of live cells and using an EM-CCD camera. Scale bar = 5 μm. Images reproduced from [120].

The oncogenic addiction of some NSCLC tumours to dysregulated EGFR signalling underpins the rationale for treating the disease by using TKIs to stop the p-EGFR-dependent downstream signalling pathways that are essential to sustain uncontrolled cell proliferation, thereby inducing programmed cell death [110]. Early experiments in lung adenocarcinoma A549 cells [111] showed that termination of p-EGFR signals by gefitinib resulted in phosphorylation and activation of the cell cycle regulator protein p53 (Box 1), followed by p53-dependent upregulation of PUMA, a pro-apoptotic, BCL2 homology 3 (BH3) domain-containing member of the BCL2 family [112], which activates rapid induction of the caspase-dependent intrinsic apoptosis pathway (reviewed in [113]) (Figure 3). Gefinitib also upregulated pro-apoptotic Fas and downregulated the anti-apoptotic proteins survivin and XIAP [111]. Further experiments in TKI-sensitive lung adenocarcinoma cell lines (PC-9 and H1560, which express the D746–750 deletion EGFR mutant, and H1975 that express the L858R mutant) showed that erlotinib dramatically induces the expression of BIM, another pro-apoptotic BH3-only member of the BCL2 family [112], which, like PUMA, also mediates TKI-induced apoptosis via the intrinsic pathway of caspase activation [114] (for a transcriptional profiling of NSCLC cell lines, see [115,116]). In cells with activating EGFR somatic mutations, BIM’s pro-apoptotic effects are synergistic with the loss of survivin, whose downregulation enhances gefitinib-induced apoptotic death in TKI-sensitive NSCLC cells [117]. These results were confirmed in lung tumours and xenografts from mice bearing mutant EGFR-dependent lung adenocarcinomas, which also display increased concentrations of BIM after erlotinib treatment [27]. Gefitinib and erlotinib also block EGFR phosphorylation of ERK and AKT, therefore pushing the closely regulated equilibrium maintained by the BH3-only BCL2 family towards the activation of effector members BAK and BAX, which thereby form oligomers at the outer mitochondrial membrane, leading to mitochondrial outer membrane permeabilisation (MOMP) and apoptosis [118,119] (Figure 3). Confocal microscopy images in live cells of the cellular distribution of BAX fused to GFP before and 3 h after stimulating apoptosis via treatment with staurosporine [120] are also shown in Figure 3.

### 2.3. The Development of Resistance to TKI Treatment

Even among the NSCLC patients that respond, the effects of gefinitib and erlotinib are transient (mean progression-free survival of 10–14 months) (reviewed in [124]). Approximately 50% of NSCLC cases develop a secondary point substitution in exon 20 of the *EGFR* gene (T790M), which confers resistance to first-generation TKI by impeding the inhibition of receptor phosphorylation through a substantially increased affinity of the EGFR’s kinase domain pocket for ATP [125]. New generations of TKIs have been developed in a race to overcome the effects of the single T790M and double L858R/T790M mutations, including second-generation (irreversible) afatinib and dacomitinib, and third-generation (T790M selective) osimertinib, which are currently used in the clinic (reviewed in [126]). However, further mutations in the *EGFR* gene and of downstream effectors eventually allow tumours to overcome the TKI therapeutic block and resume uncontrolled proliferation [6,127]. 

Acquired mutations not only involve the *EGFR* gene (e.g., the secondary T790M acquired *EGFR* mutation and others [26]), but can also be EGFR independent (e.g., loss of p53 function, constitutive activation of RAS, etc. (Box 1)). Together, acquired mutations contribute to increase tumour heterogeneity and develop pro-survival adaptation mechanisms at cellular and tumour levels [128,129]. However, for such mutations to accumulate, cells need to first survive the initial therapeutic insult. An important observation is that TKIs fail to trigger apoptosis in a fraction of responsive NSCLC tumour cells addicted to EGFR signals, instead inducing G1 cycle arrest [27]. Whilst the latter contributes to suppress tumour growth, quiescent cells surviving TKI treatment have the opportunity to acquire mutations and/or invoke adaptation mechanisms by which they can eventually resume uncontrolled proliferation. Chiefly among EGFR-dependent mechanisms of adaptation are pro-survival functions exercised by EGFR independently of its kinase activity, which can be recapitulated in quiescent cells by the actions of TKI-bound EGFRs (reviewed in [39]). Examples of EGFR kinase-independent functions include stimulation of DNA synthesis [130], expression of the c-fos proto-oncogene [131], and dysregulation of cellular self-degradation processes (reviewed in [132]), with the latter extensively imaged by fluorescence microscopy methods, and discussed below. 

## 3. At the Interface between Autophagy and Resistance to TKIs 

### 3.1. A Brief Overview of (Macro) Autophagy 

**Figure 4:**
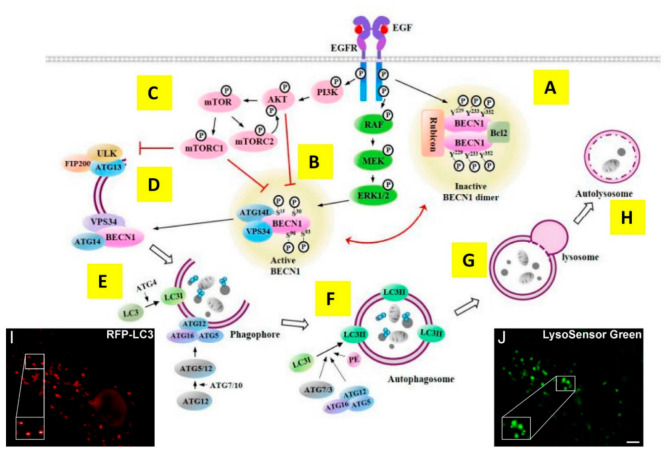
EGFR-dependent regulation of autophagy. (**A**) Tyrosine phosphorylation of Beclin1 by EGFR leads to homodimerisation of Beclin1 and subsequent binding of inhibitors of autophagy, such as Rubicon and B-cell lymphoma 2 (Bcl-2), to Beclin 1 to inhibit autophagic activity. (**B**) EGFR-PI3K/AKT signalling [139] can downregulate autophagy via phosphorylation of Beclin 1 on serine residues. (**C**) AKT phosphorylates mTOR [144], which forms two multiprotein complexes, mTORC1 and mTORC2, the former sensitive to nutrients and the latter regulated via PI3K and growth factor signalling [145]. (**D**) Phosphorylated mTORC1 inhibits the interaction between unc-51-like kinase (ULK), class III PI3-kinase (VPS34), and family-interacting protein FIP200 (the ULK complex), which drives at the endoplasmic reticulum (ER) the nucleation of the phagophore [146], the precursor double-membrane structure of the autophagosome [147,148]. (**E**) Downstream of the ULK complex, the formation of the Beclin 1 (BENC1)-containing class III PI3 kinase complex, consisting of Beclin 1 (BECN1), the lipid kinase vacuolar protein sorting 34 (VPS34), and ATG14, leads to its recruitment to the nascent phagophore to promote its elongation. (**F**) Formation of the autophagosome is executed by the sequential function of the autophagy-related (ATG) proteins (reviewed in [149]). This requires two ubiquitin-like protein conjugation systems, ATG12 and protein light chain 3 (LC3), which between them involve one protease, ATG4, which cleaves microtubule-associated protein 1-light chain 3 (LC3) at its carboxyl terminus, the E1-like enzyme ATG7 (common to both conjugation systems), the E2-like enzymes ATG10 (ATG12 system), and ATG3 (LC3 system). ATG4 cleavage of LC3 at the C-terminus results in the formation of LC3I, which is conjugated with phosphatidyl ethanolamine (PE) to become LC3II by the action of a complex between ATG12-ATG5-ATG16L1 [56]. LC3II is present on autophagosomes, and protein fusions of LC3II and fluorescence proteins are used to quantify the autophagic flux [58]. (**G**) Autophagosomes fuse with lysosomes to form autolysosomes (**H**) where intracellular contents are degraded [150]. Autophagosomal cargo (organelles or proteins) is recognised by being marked with Lys63-linked ubiquitin chains that interact with adaptors, including sequestosome 1 (SQSTM1/p62), which specifically interact with LC3-like proteins, thus targeting the cargo to autophagosomes [151]. (**I**) Fluorescence live cell image of exogenous RFP-LC3 in Purkinje neurons displaying typical puncta that reports the formation of autophagosomes, which can be quantified [60]. (**J**) Same area and scale as in (**I**) showing lysosomes labelled with Lysosensor Green. Images were taken under a wide-field microscope using a deep-cooled CCD camera. Scale bar = 5 μm [60]. Main figure reprinted from [36]. (**I**,**J**) reprinted from Methods Enzymol 453, “Live-cell imaging of autophagy induction and autophagosome-lysosome fusion in primary cultured neurons” 2009, 145–158, with permission from Elsevier [60].

Macroautophagy (here referred to as autophagy) is an evolutionary conserved, tightly regulated cellular self-degradation process. Derived from Greek “self” and “eating”, baseline (constitutive) autophagy occurs in normal cells under physiological conditions. The housekeeping job of autophagy is to remove unwanted old/misfolded proteins, defective endoplasmic reticulum areas, and damaged organelles, and to eliminate intracellular pathogens (reviewed in [133]). Cargo degradation is accomplished by engulfing identified portions of the cytoplasm containing selected cargo (mediated by cargo ubiquitination and recognised by the sequestosome 1 (SQSTM1/p62) that targets the ubiquitylated cargo to autophagosomes for degradation [134]) into double-membrane vesicles called autophagosomes, which fuse with lysosomes to catabolise their contents [135]. The key players in this process are the autophagy-related (ATG) proteins [136] (Figure 4).

Via its energy sensing function, mTOR is the cell’s autophagy master downregulator, promoting anabolic processes (e.g., biosynthesis of proteins, lipids, and organelles) and limiting catabolic processes, such as autophagy (reviewed in [137]). When glucose, amino acids, and/or growth factors are abundant, activation of the PI3K-AKT-mTOR signalling cascade leads to mTOR phosphorylation (p-mTOR) [105]. Under starvation, the accumulation of unphosphorylated mTOR triggers autophagy as part of the initial cell’s pro-survival response (reviewed in [138]). Independently of mTOR, activated AKT [139] and ERK [140] can inhibit or induce autophagy, respectively, via phosphorylation at serine residues of the essential autophagy protein Beclin 1, a protein encoded by the BECN1 gene, the mammalian homolog of yeast Atg6, and a key component of the autophagic process and at the crossroads of apoptotic signals (reviewed in [141]). Beclin 1 (BENC1) phosphorylation at tyrosine residues by EGFR leads to the inhibition of its key function at the centre of autophagy upregulation (reviewed in [142]) (Figure 4A). 

Given that the activation of EGFR signalling tends overall to inhibit autophagy (Figure 4B,D), gefitinib and erlotinib inhibition of EGFR signalling was unsurprisingly found to induce autophagy in a dose-dependent manner and in multiple cancer cells of multiple origins [143] (Figure 4E,H)). As outlined in Box 2, the most common method to image autophagy is to use wide-field or confocal microscopy to detect FP-LC3 puncta, which report the presence of autophagosomes (Figure 4I). The colocalisation of FP-LC3 with lysosomal markers, in turn, reveals the formation of autolysosomes (Figure 4J).

### 3.2. Autophagy and Its Relationship with Apoptosis and Cancer Progression

Autophagy plays a paradoxical role in cancer [152]. In the early stages of the disease, autophagy can delay tumour progression by removing aberrant cell structures, such as, for example, cytoplasmic DNA-containing micronuclei, which enable cancer cells to accelerate changes in their chromosomal architecture [153]. In advanced stages, autophagy can antagonise apoptosis in two ways. On the one hand, it can remove damaged mitochondria [154] (a common “off-target” effect of environmental toxins, such as TKIs [155]). This eliminates the metabolic stresses accumulated in these damaged mitochondria from overproduction of reactive oxygen species (ROS), thereby inhibiting the pro-apoptotic function of the latter (reviewed in [156]). On the other hand, by catabolising the autophagosomal cargo, autophagy can provide cancer cells with additional energy and nutrients to survive periods of acute stress and avoid programmed cell death (reviewed in [157]). 

Autophagy and apoptosis share key regulators, including EGFR downstream signalling effectors (reviewed in [158]). Downstream of JAK and c-SRC, STAT3 signals also play an important role in the regulation of autophagy (reviewed in [159]) (Figure 2B). Phosphorylated STAT3 translocates from the cytoplasm, where it inhibits autophagy (e.g., by interacting with transcription factors, such as FOXO1/3) to the nucleus, where it upregulates autophagy-suppressing genes, including the BCL2 family, which function at the crossroad between intrinsic apoptosis and autophagy regulation [112]. Activated p-STAT3 can also translocate to mitochondria, where it suppresses autophagy-stimulating mitochondrial ROS production [159], with the latter also being at the intersection between autophagy and intrinsic apoptosis. Inhibition of STAT3 stimulates autophagy in vitro and in vivo [160]. 

### 3.3. The Dual-Edged Sword of Targeting Autophagy 

Given that autophagy can promote survival under stress conditions, its targeting has emerged as a potential mechanism to overcome TKI resistance (reviewed in [36]). In cancer cells where TKIs induce cytoprotective autophagy, a body of evidence suggests that inhibiting autophagy can lead to the restoration of TKI-induced apoptosis. As an example, Han et al. [161] showed in NSCLC cell lines (A549, H1299, H292, H1650, and SK-MES-1) that gefitinib and erlotinib induce autophagy via inhibition of the PI3K/AKT/mTOR signalling pathway, and that the cytotoxicity of these TKIs was greatly enhanced after autophagy inhibition via chloroquine, a chemical that inhibits autophagic flux by decreasing autophagosome-lysosome fusion [162]. Also using NSCLC cell lines (A549, H322, H358, and H460), Zou et al. [59] showed that the survival of erlotinib-resistant cells was impaired when TKI was combined with chloroquine. The suggested therapeutic benefit of inhibiting autophagy has also been supported by some clinical trials in which autophagy was targeted in NSCLC tumours in combination with TKIs (reviewed in [152]). 

Recapitulating the paradoxical role of autophagy in cancer, inducing autophagy can instead reinstate sensitivity to TKI-induced apoptosis in some settings (reviewed in [163]). As an example, work in wtEGFR-expressing cancer cells highly resistant to EGFR-TKIs (Hela-R30 and OSCC 686LN), in which autophagy is not robustly activated, showed that rapamycin, an mTOR inhibitor, both restored autophagy in these cells and augmented the cytotoxic effect of EGFR-TKIs [143]. These results were backed in vivo by the observation that changes in autophagic activity are associated with inverse changes in the rates of tumour growth of NSCLC xenografts expressing an activated EGFR mutant [164].

If both autophagy inhibitors and inducers can be useful in combination with EGFR TKIs in treating EGFR-driven NSCLC tumours, it must be concluded that another regulatory process upstream of autophagy must be responsible, at least in part, for some cancer cells to be more dependent on autophagy than others. Upstream of autophagy is endocytosis, a process that regulates the compartmentalisation of EGFR at the plasma membrane and intracellular vesicles and organelles. From these compartments, EGFR can orchestrate autophagy not only by regulating its cognate signalling cascades, but also via direct interaction with key proteins of the autophagic regulatory machinery.

## 4. Endocytosis and Autophagy: Friends or Foes?

### 4.1. Endocytosis Walks ‘Hand-In-Hand’ with Autophagy

Endocytosis is an evolutionarily conserved, tightly regulated cellular function that also plays a key role in cancer (reviewed in [165]). Involving, like autophagy, a lysosome-mediated degradation process, endocytosis also shares with autophagy the intracellular membrane trafficking machinery jointly coordinated by the endoplasmic reticulum, endosomes, and lysosomes (reviewed in [166]). Cells orchestrate endocytosis to rapidly internalise selected (e.g., ubiquitylated) integral plasma membrane regions and their cargo, which is then trafficked through endosomal vesicles (reviewed in [167]). Major oncogenic drivers (such as p53, RAS, *EGFR* duplications and mutations) and oncogenic signals (e.g., from activated ERK and AKT) cooperate to dysregulate endocytic trafficking (reviewed in [165]). 

### 4.2. Clathrin-Mediated Endocytosis Distributes Egfr throughout Vesicles and Organelles 

**Figure 5:**
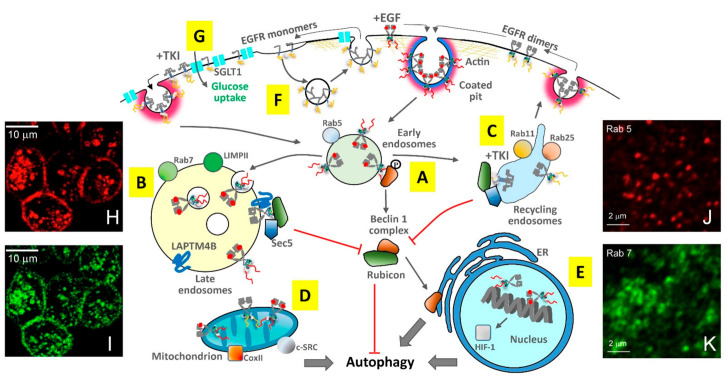
Interdependence between autophagy and endocytosis. (**A**) At early endosomes marked by Rab5, phosphorylation of Beclin 1 by EGFR [164] leads to the formation of the autophagy-inhibitory complex between Beclin 1 and Rubicon [170]. (**B**) When trafficked to late endosomes marked, for example, by Rab7 and/or LIMPII, which is also a lysosomal marker, EGFR forms a protein complex with late endosome resident LAPTM4B and the exocyst component Sec5 to recruit the Beclin 1-antagonist Rubicon [171], thereby blocking the formation of the Beclin 1 complex with Rubicon, and thus allowing Beclin 1 to be translocated to the ER, where it can form the Beclin 1-containing class III PI3 kinase complex to promote the elongation of the autophagophore and induce autophagy. (**C**) When EGFR is bound to TKI, recruitment of Rubicon proceeds recycling endosomes marked by Rab11 and Rab25, requiring Sec5 but not LAPTM4B [171]. (**D**) Phosphorylation of CoxII at the mitochondria by EGFR and c-SRC changes mitochondrial metabolism, leading to the induction of autophagy [172]. (**E**) In the nucleus, EGFR upregulates the transcription of HIF-1, leading to the induction of hypoxia-mediated autophagy [173]. (**F**) At the plasma membrane, inactive receptors (not bound to EGF) can be basally endocytosed. (**G**) Ligand-free, kinase inactive EGFR can interact via their ectodomains with the sodium/glucose cotransporter 1 (SGLT1) in the presence and absence [174]. (**H**) EGFR bound to Texas red-EGF is transported to late endosomes/lysomes of PC9 cells marked by immunofluorescence staining of LIMPII (**I**) [50] (Bars in (**H**,**I**) are 10 μm). (**J**,**K**): Immunofluorescence microscopy images of Rab5, Rab7, and LAMP-1-marked endosomes in A549 cells [44]. The organic dyes used are Alexa Fluor 488 nm, Alexa Fluor 555 nm, and Alexa Fluor 647 nm. Images were taken using a laser scanning confocal microscope. (Bars in J and K are 2 μm) (**H**,**I**) reprinted by permission from Springer Nature Customer Service Centre GmbH: Springer Histochem Cell Biol Nishimura, Y.; Bereczky, B.; Ono, M. 2007 [50]. (**J**,**K**) reprinted from Heliyon 5, e02375, Shearer, L.J.; Petersen, N.O. “Distribution and Co-localization of endosome markers in cells”, 2019, with permission from Elsevier [44].

Growth factor stimulation triggers EGFR endocytosis via clathrin-dependent and clathrin-independent pathways (reviewed in [168]). More extensively studied in the context of TKI-resistance, the clathrin-dependent endocytosis of EGF-bound EGFR complexes is followed by endosomal trafficking and either sorting to recycling endosomes to be trafficked back to the plasma membrane or sorting for degradation in proteolytic lysosomes (reviewed in [169]) (Figure 5).

For EGF-EGFR complexes to be sorted for degradation, their ubiquitination, which follows phosphorylation, is required [175]. Ubiquitination is accomplished by EGFR’s C-terminal interaction with the E3 ubiquitin-protein ligase Cbl (reviewed in [176]). Ubiquitylated EGFR is sequentially transported to early and late endosomes, characterised, respectively, by the formation of transient assemblies of the small Rab GTPases Rab5 and Rab7 (Figure 5A,B). At late endosomes, EGFR is dephosphorylated by endoplasmic reticulum phosphatases. EGFR degradation requires interactions with the Retromer and the ESCRT complexes at multivesicular bodies (MVBs), which fuse with lysosomes (reviewed in [168]). EGF-EGFR complexes sorted for recycling to the plasma membrane are instead trafficked from early endosomes to Rab11 and Rab25 recycling endosomes (reviewed in [177]) (Figure 5C). Excessive recycling contributes to the accumulation of receptors at the surface, which results in signal amplification and can lead to uncontrolled cell proliferation (reviewed in [178]). 

Through less well-understood mechanisms, clathrin-mediated endocytosis and endosomal sorting are also involved in the transport of EGF-EGFR complexes to the close vicinity of the mitochondrial outer membrane [172] (Figure 5D), especially in highly invasive NSCLC cells [179], and to the inner nuclear membrane and the nucleoplasm [180], where EGFR functions as a co-transcription factor (Figure 5E). Nuclear-localized EGFR is highly associated with disease progression and a worse overall survival in numerous cancers, and with enhanced resistance to anti-EGFR TKIs (reviewed in [181]).

In the absence of growth factor stimulation, EGFR can also be constitutively endocytosed. The endocytosis of EGF-free wtEGFR occurs at a slow basal level, and the receptors are recycled back to the plasma membrane [182] (Figure 5F). (These receptors also interact with the sodium/glucose cotransporter 1 (SGLT1) [174] (Figure 5G), as discussed below). Constitutively activated, EGF-free EGFR mutants (e.g., L858R, D746–750, and T790M) display a significantly higher basal endocytosis [182]. However, because of their defective association with c-Cbl and ubiquitinylation, these activated EGFR mutants do not progress to late endosomes, MVBs, or lysosomes, accumulating instead in recycling endosomes, from where they traffic through endocytic recycling compartments back to the plasma membrane [183] (Figure 5C). The aberrant constitutive endocytosis of activated EGFR mutants not only confers enhanced signalling activity, but also promotes their colocalisation and association with c-SRC, thereby amplifying signalling dysregulation and contributing to tumour progression [184]. 

### 4.3. Endocytic Trafficking Underpins the TKI Response 

Because of its dual role in terminating or amplifying EGFR signalling, much effort has been devoted to investigating the role of endocytosis in cancer cell survival and resistance to TKI therapy (reviewed in [165]). In the search for endocytic properties that may be exploited to predict responsiveness to gefitinib, Nishimura et al. [50] used confocal immunofluorescence microscopy to compare the endolysosomal distribution of fluorescent EGF-EGFR complexes in gefitinib-sensitive NSCLC PC9 cells expressing the D746–750 EGFR mutant and gefitinib-resistant QG56 cells expressing wtEGFR. In both cell lines, EGF-induced EGFR endocytosis was evidenced by the appearance of characteristic punctate endosomal vesicles loaded with fluorescent EGF-EGFR complexes (Figure 5H)). However, only gefitinib-sensitive expressing cells displayed efficient EGF-induced targeting to lysosomes immunostained with antibodies against the late endosomal/lysosomal marker LIMPII (Figure 5I) (fluorescence images of other endosomal markers, e.g., Rab5 and Rab7, can be found in Figure 4J,K). 

In TKI-responsive cells, Nishimura et al. [50] found that endocytosis was inhibited by gefitinib binding, and that the small fraction of receptors that could still internalise were sorted for recycling. Conversely, in gefitinib-resistant cells, EGF-bound wtEGFR did not efficiently progress beyond early endosomes, and gefinitib binding did not inhibit endocytosis in these TKI-resistant cells (these endocytic differences between TKI-sensitive and TKI-resistant cells were recently confirmed [185]). The protein SNX1, a component of the Retromer and part of the trafficking/sorting machinery that targets EGFR to the lysosomes, was found to negatively regulate EGF-dependent EGFR trafficking from early endosomes to late endosomes/lysosomes, and inhibition of SNX1 was shown to underpin TKI resistance [186]. 

To investigate the potential effects of endocytosis in the intrinsic resistance to TKIs displayed by ~80% of NSCLC patients with tumours driven by wtEGFR overexpression, Jo et al. used wide-field immunofluorescence microscopy to compare the endocytic trafficking of a fluorescent derivative of EGF bound to wtEGFR in NSCLC-derived gefitinib-sensitive H358 cells and gefitinib-resistant H1703 cells [40]. EGF-induced wtEGFR endocytosis was detected in both gefitinib-sensitive and -resistant cells in a clathrin-dependent fashion [40,185]. Only in TKI-sensitive cells were EGF-wtEGFR complexes trafficked beyond early endosomes into recycling endosomes. Furthermore, the expression of Rab25 was implicated in TKI sensitivity because its knockdown reduced the pro-apoptotic effect of the TKI [40]. 

### 4.4. Compartmentalised EGFR Interactions Balance the Regulation of Autophagy 

Besides indirect regulation by EGFR of the autophagic process via signalling pathways (Figure 4B,D), direct interactions between EGFR and proteins of the autophagy machinery can occur in different endocytic compartments throughout the cell (Figure 5). By altering the localisation and distribution of EGFR in different cellular compartments [187], the dysregulation of endocytosis in cancer cells can therefore have a profound effect on EGFR’s regulation of the autophagic process (reviewed in [188]). 

Direct regulation of autophagy by EGFR can be dependent or independent of the receptor’s kinase activity. Weihua et al. [174] ascertained that loss of wtEGFR expression at the plasma membrane of PC-3MM2 prostate cancer cells triggers autophagy via a decrease in intracellular glucose but that basal glucose levels were maintained if cells bearing wtEGFR were treated with the anti-EGFR reversible TKI AEE788 [189]. Through EGFR upregulation of basal glucose levels, cells avoid mTOR-mediated autophagic cell death [190]. The EGFR-dependent (but kinase-independent) mechanism was the interaction between the ectodomain of EGFR and the sodium/glucose cotransporter 1 (SGLT1) [191], which occurs at the plasma membrane (Figure 5G). 

Another EGFR-dependent but kinase-independent mechanism was described by Tan et al. [171], who used wide-field immunofluorescence microscopy to show that whilst the loss of EGFR expression inhibited autophagy in MDA-MB-231, HeLa, A431, and HEK293 cancer cells, re-expression of a kinase-dead mutant EGFR (K745A) rescued autophagy via EGFR’s interaction with the late endosome/lysosomal marker LAPTM4B [192] and the recruitment by the EGFR/LAPTM4B complex of the exocyst Sec5 component [193] (Figure 5C). The EGFR/LAPTM4B/Sec5 complex recruits Rubicon, disrupting its inhibitory interaction with Beclin 1 and initiating autophagy [170] (Figure 5C). In TKI-responsive cells, gefinitib-bound EGFRs dispense of LAPTM4B to bind Rubicon, only requiring Sec5, which, unlike LAPTM4B, also localises to recycling endosomes where gefitinib-bound EGFR accumulate [171] (Figure 5B).

As also outlined above, a kinase-dependent function elicited by EGFR at early endosomes was reported by Wei et al. [164], who also used wide-field immunofluorescence microscopy to show in HeLa cells and NSCLC cell lines (A549, HCC827, and H1975) that both EGF-bound wtEGFR and EGF-free activated EGFR mutants (L858R and D746–750) interact with Beclin 1 at early endosomes (Figure 5B). Via this interaction, Beclin 1 becomes phosphorylated in multiple tyrosine residues, forming dimers that recruit its antagonist Rubicon, and thereby inhibiting autophagy in a fashion independent of mTOR (Figure 4 and Figure 5B). In TKI-responsive cells, gefitinib blocks the Beclin 1–EGFR interaction, thereby restoring autophagy [164] (Figure 4 and Figure 5D). 

Fluorescence microscopy also played a crucial part in uncovering another kinase-dependent mechanism of autophagy regulation by EGFR, which is elicited by mitochondrial EGF-bound p-EGFR, which reaches the mitochondria via clathrin-mediated endocytosis to phosphorylate cytochrome c oxidase subunit II (CoxII) [172], an enzyme in the mitochondrial electron transport chain at the heart of the mitochondrion-dependent intrinsic apoptotic signalling [194] (Figure 5E). c-SRC translocates to mitochondria alongside EGFR, and also phosphorylates CoxII [172] (Figure 5E). Phosphorylation of CoxII reduces ATP production [172], facilitating the conversion from predominantly oxidative phosphorylation to glycolysis and pentose metabolism [195]. These changes in mitochondrial metabolism promote autophagy and contribute to cell resistance to extreme conditions by inducing quiescence [196,197]. 

Fluorescence imaging, and in particular the FISH assay, has also played a crucial role in investigations of autophagy regulation by nuclear translocated EGFR in NSCLC [55]. EGF-bound p-EGFR upregulates at the nucleus the transcription of pro-survival/adaptation proteins, a key one being the hypoxia-inducing factor (HIF-1), which regulates hundreds of genes to allow adaptation to moderate to severe hypoxia (∼3–0.1% oxygen), which occurs at the later stages of tumour growth when partaking cells find themselves separated from the local vasculature (reviewed in [173]) (Figure 5F). Oxygen-sensing by HIF-1 is an important positive regulator of cytoprotective autophagy via its upregulation of two BH3-only proteins, BNIP3 and its homologue BNIP3L, which are overexpressed in hypoxia and cooperate to induce autophagy via disruption of the inhibitory interaction between Bcl-2 and Beclin 1 (reviewed in [197]). An inhibitor of HIF-1α, YC-1, was found to significantly inhibit the cytoprotective autophagy induced by gefitinib by disrupting the fusion of autophagosomes and lysosomes, thus increasing the pro-apoptotic effect of gefitinib in gefitinib-resistant NSCLC cells [198]. 

### 4.5. Endocytosis Underpins Different Responses to TKIs Depending on the EGF Stimulus

In TKI-resistant NSCLC-derived cell lines (H1703 and SNU-1327 expressing wtEGFR, and H1975, which harbour the double L858R/T790M mutation), the inhibition of clathrin-mediated endocytosis of EGF-EGFR complexes (e.g., via dynasore [40], Filipin III [185], or Pitstop [199]) resulted in a marked increase in the fraction of TKI-induced apoptotic cells [40]. In TKI-resistant cells expressing the double L858R/T790M mutant, besides restoring TKI-induced apoptosis, clathrin inhibition also restored receptor degradation via a macropinocytosis-dependent lysosomal pathway associated with loss of mutant-EGFR-dependent signalling via p-AKT and p-ERK [199]. Consistent with results in cells, combination treatment of gefitinib and the clathrin inhibitor PAO resulted in tumour regression accompanying apoptosis in xenograft mouse models [185].

Conversely, without the EGF stimulus, De Wit et al. in contrast found that endocytosis was associated with increased sensitivity to TKIs [200]. High-throughput confocal microscopy was used to compare TKI-refractory eGFP-wtEGFR with other 10 eGFP-tagged EGFR mutation constructs for which their responsiveness to gefitinib was documented. All constructs were stably expressed in Hela cells. The results revealed that TKIs, which remain associated with EGFR after its dephosphorylation, strongly induced intracellular accumulation of EGFR in cells expressing TKI-responsive activating mutations. De Wit et al. showed that internalisation of TKI-bound EGFR predicts the degree of cytotoxicity of EGFR TKIs, thereby mimicking clinical efficacy. 

## 5. A Perspective from a Structural Viewpoint 

Although still in need of further development, the combination of standard fluorescence microscopy and super-resolution methods has already made some inroads in providing a structural perspective of TKI function (Box 2). Early results showed that gefitinib and erlotinib induce the dimerisation of EGFR at the plasma membrane [86,201]. Using multiphoton confocal FLIM in combination with FRET and biochemical covalent cross-linking analyses, Bublil et al. [202] proposed that gefitinib and erlotinib target the active conformation of EGFR’s kinase domain (erlotinib was later suggested by X-ray crystallography to bind both inactive and active conformations [203]). Electron microscopy showed that gefitinib and erlotinib binding induces the formation of so-called ‘quasi-dimers’ in which two TKI-bound kinases form a structure akin to the catalytically active asymmetric kinase dimer that forms through growth factor binding [204] (Figure 2A). 

**Figure 6:**
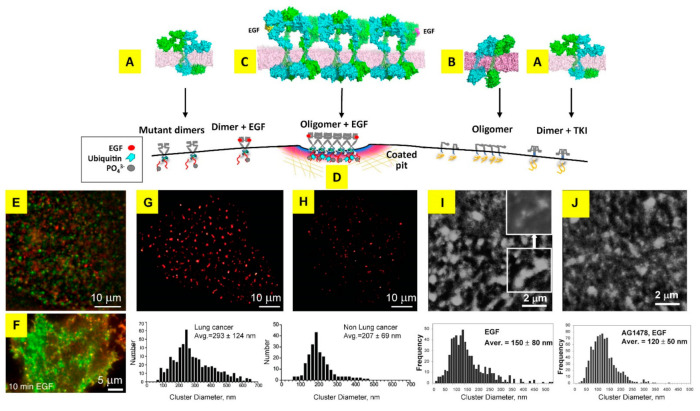
Structure–function relationships at the plasma membrane. (**A**) Stalk-to-stalk ectodomain dimers structurally coupled to an active asymmetric kinase dimer structure are formed by EGFR dimers bearing activating kinase mutations [86]. In the presence of bound TKIs, wtEGFR adopts the same conformation in the absence of bound EGF. (**B**) In the absence of bound EGF, wtEGFR forms inactive polymers mediated by a repetition of head-to-head ectodomain interactions. (**C**) In the presence of bound EGF, EGFR forms oligomers via a face-to-face interaction that outcompetes EGF binding [85]. EGF can therefore only bind the two EGFR molecules at the ends of the oligomer. Another face-to-face interaction mediating oligomer formation has also been reported (not shown) [206]. (**D**) Oligomers colocalised to nascent coated pits where their signalling is amplified [207]. Cbl-dependent ubiquitination is required for progression of EGFR oligomers into clathrin-coated pits [208]. (**E**) Merged time-integrated SPT-FRET donor (EGF-Cy3; green) and acceptor (EGF-Cy5; red) images of a sample of A431 cells exposed to 0.25 nM EGF-Cy3 and 0.5 nM EGF-Cy5 that were labelled with wtEGFR and collected using TIRF illumination. (**F**) A single frame of a dual-colour SPT time-series acquired after 10 min of stimulation with 16 nM EGF in an MCF-7 cell expressing EGFP-PTB (green) and SNAP-EGFR labelled with Cy3 (red). Reconstructed dSTORM images of Alexa647-Cetuximab-labelled wtEGFR in the plasma membrane of lung cancer cells (**G**) and normal lung epithelial cells (**H**). The histograms below show the size distribution of EGFR clusters on the cell surface. NSOM fluorescence images of EGF-treated HeLa cells in the absence (**I**) and presence (**J**) of TKI (AG1478). Results show that wtEGFR localises in small clusters with a range of sizes and intensities, quantified in the histograms below. The inset at the top right of (**I**) shows the region outlined at the bottom right on a different intensity scale, illustrating that some brighter features are multiple small clusters. (**E**) reprinted from Biophys J 94, 803–819, Webb, S.E.; Roberts, S.K.; Needham, S.R.; Tynan, C.J.; Rolfe, D.J.; Winn, M.D.; Clarke, D.T.; Barraclough, R.; Martin-Fernandez, M.L. “Single-molecule imaging and fluorescence lifetime imaging microscopy show different structures for high- and low-affinity epidermal growth factor receptors in A431 cells” 2008, with permission from Elsevier. (**F**) reprinted from [207]. (**G**,**H**) reprinted from [209]. (**I**,**J**) reprinted by permission from Springer Nature: Springer, Cell Research, “Regulation of EGFR nanocluster formation by ionic protein-lipid interaction” Ye Wang et al., 2014 [209].

A significant part of the challenge in ascertaining any structural dimension of TKI responsiveness is methodological. Sufficient resolution (few nanometres) is hard to achieve deep within the cell but somewhat easier to attain at the plasma membrane, for example, by using TIRF microscopy in tandem with single particle localisation methods (Box 2). A method that can achieve sub-5 nm resolution is FLImP [85], which combined with FRET allowed Zanetti-Domingues et al. to find that the intracellular asymmetric kinase dimers induced by erlotinib binding were not just quasi-dimers, but rather that the intracellular asymmetric kinase dimer was structurally coupled across the plasma membrane to the formation of a stalk-to-stalk ectodomain dimer [86] (Figure 6A). FLImP and FRET results were combined with long-duration MD simulations to derive the atomic resolution structures shown in Figure 6A,C. Possibly of relevance to the heterogeneous clinical response of NSCLC patients, before binding TKIs, the packing arrangements of constitutively activated EGFR mutants and wtEGFR are rather different. Activated EGFR mutants (e.g., L858R) mostly form stalk-to-stalk ectodomain dimers (Figure 6A) whilst inactive wtEGFR forms larger basal oligomers involving an extracellular head-to-head interaction (Figure 6B), which can keep the receptor autoinhibited in the absence of EGF by inhibiting the formation of the catalytically active asymmetric kinase dimer [86,96,204,205]. TKI binding breaks the basal oligomers formed by wtEGFR [86] whilst activated EGFR mutants retain the stalk-to-stalk dimer conformation [204].

Coban et al. [210] used single particle tracking (SPT) in combination with FRET (Box 2) to probe interactions between two EGFR molecules in TKI-bound EGF-EGFR dimers. The results revealed that gefitinib further stabilises the EGF-EGFR dimer (an example of a fluorescence SPT image in combination with FRET is shown in Figure 6E). One can envisage that a TKI-induced increase in dimer stability would potentially alter the tight spatiotemporal control by which endocytosis and/or autophagy regulate TKI responsiveness, as found in other contexts for growth factor-bound EGFR in the absence of TKI (see, for example, [211]). 

EGFR dimerization is a key event for receptor activation, but the role of higher-order EGFR oligomers and clusters, first revealed by the pioneering work of Clayton et al. [212], is less well understood. In the absence of bound TKIs, two structures of EGF-bound EGFR oligomers have been reported, based on repeating ectodomain face-to-face interactions that involve the EGF binding site or side-to-side interactions [85,206] (the atomic-resolution structure of oligomers formed by face-to-face interactions derived from MD simulations is shown in Figure 6C). The two oligomer types, which were predicted to display a different intracellular arrangement of kinase domains, where shown to organize kinase-active dimers in ways optimal for auto-phosphorylation in trans between neighbouring dimers, and/or display cooperative activation between the kinase domains, thereby boosting C-terminal tail phosphorylation. 

Linking EGFR oligomerisation with clathrin-mediated endocytosis, Ibach et al. [207] used two colour SPT (Figure 6F) to investigate the formation of large wtEGFR clusters and their mobility at the plasma membrane. Results showed that in the immobile state, EGFR clusters associate in clathrin-coated pits (Figure 6D). The latter was a requirement to amplify EGFR phosphorylation, leading to the formation of local gradients of signalling active receptors. Inhibiting clathring-mediated endocytosis using dynasore substantially delayed the onset of signal activation.

Highlighting differences in EGFR aggregation in normal and cancerous cells, Wang et al. used STORM (Box 2) to characterise the clustering profile of wtEGFR at the plasma membrane of freshly isolated lung cancer epithelial cells and their paired normal lung cells [209] (Figure 6G,H). Results showed that wtEGFR forms nanoclusters at the plasma membrane of both normal and lung cancer cells, but the number and size of these clusters is significant larger in EGFR-overexpressing cancer cells (~300 nm v ~200 nm dia). Interestingly, cluster formation depended on interactions between EGFR and PIP_2_, a plasma membrane phospholipid that is catalysed by PI3K into PIP_3_, the substrate for AKT [100]. 

The clustering profile of EGFR is changed by its interaction with TKIs. Abulrob et al. [213] employed NSOM (Box 2) to examine the nanoscale clustering of wtEGFR in HeLa cells and the influence of the ATP-competitive tyrosine phosphorylation inhibitor, Tyrphostin (AG1478) [214] (Figure 6I,J). Tyrphostin has analogous functionality to gefitininib and erlotinib but is not approved in the clinic. Results from NSOM revealed that wtEGFR is organized in clusters of an average diameter of ~150 nm at the plasma membrane, with the numbers of receptors in individual clusters varying from a few to >100. Tyrphostin increased the cluster density and the fraction of clusters with smaller diameters and fewer receptors, resembling the pattern of EGFR clustering in normal cells [209]. Tyrphostin also decreased the fraction of EGFR that colocalizes with rafts [213]. This is an interesting observation because EGFR colocalisation with lipid rafts is correlated with resistance to gefitinib in breast cancer cell lines expressing wtEGFR, and disrupting rafts by depleting plasma membrane cholesterol with lobostatin was found to re-sensitise these resistant cell lines to EGFR-TKIs [215]. Moreover, p-Akt, which persisted in resistant cell lines oncogenically addicted to EGFR for proliferation, was abrogated by lovostatin. 

## 6. Conclusions

Fluorescence microscopy has played a pivotal role complementing the information derived from genomics and transcriptomics [216] with information that correlates key functionality with sub-cellular localisation. Beyond this, by imaging in the physiological cell context, and in the presence and absence of TKI, the combination fluorescently tagged proteins (e.g., EGFR and its signalling and regulatory effector machinery) and fluorescence microscopy methods have facilitated investigation of the crosstalk between canonical functions affected by catalytically competent EGFR with those that may be exerted by TKI-bound EGFR independently of its kinase activity. This has afforded comparisons between receptors bearing wtEGFR and TKI-sensitising and TKI-refractory activating mutations. In this way, fluorescence imaging has also helped to decipher some of the unintended consequences that the removal of kinase activity has on EGFR endocytic trafficking, and/or the regulation of pro-survival functions, revealing the significance of these changes in helping the cell to survive therapeutic insults. 

Fluorescence microscopy methods have also played a unique role in probing in the cellular context the mechanisms underlying TKI changes in receptor structure. More successfully at the plasma membrane, in combination with TIRF and super-resolution methods (e.g., STORM and NSOM), fluorescence microscopy has provided us with the tools to make inroads towards our understanding of the functional consequences of TKI-induced oligomerisation changes. These changes are likely to have a significant effect on the ability of the cell to traffic EGFR to a particular endocytic compartment, and/or on the ability of the receptor to orchestrate cellular functions, such as EGFR’s regulation of autophagy. 

Some TKI-induced changes in receptor conformation, such as those revealed by FLImP, might modulate, via still unknown mechanisms, the interactions between TKI-bound EGFR and its multiple kinase-independent targets. This is suggested by the observation that different EGFR kinase dead mutants promote different cellular functions: for example, mutant K721R-EGFR but not D813A-EGFR promotes cell survival in the absence of interleukin 3 (IL3) in 32D murine hematopoietic cells [217], mutant K721M-EGFR can stimulate the expression of c-fos [131], and D813A-EGFR but not K721M-EGFR is able to stimulate DNA synthesis [130]. These results suggest that specific kinase-inactive conformations are important for certain cell survival functions. The field is currently ripe to allow structure–function studies in which one can ask, for example, which are the TKI-bound EGFR conformations that can interact (or not) with SGLT1 to upregulate glucose and prevent autophagic-dependent cell death [174], and/or to be recognised by endocytic adaptors and/or clathrin [218]. One could also ask how the conformation of TKI-bound EGFR differs when the receptor is bound to EGF, which may conceivably go some of the way towards explaining the paradoxical effects on TKI resistance found when endocytosis or autophagy are inhibited. 

Further methodological developments are required to elucidate how TKI-induced conformational changes might orchestrate interactions deep within the cell between EGFR with the endocytic sorting machinery [219] and/or compartmentalised autophagy effectors [36], and/or how mitochondrial EGFR interacts with metabolic regulators driving resistance [220]. Of great interest are new developments in correlative light and electron microscopy [221], where the latter is poised to eventually replace the role of MD simulations in understanding structure at the atomic resolution.

The hope is that current and new super-resolution imaging methods, such as those discussed here, will make a difference in the development of novel rational and complementary therapies by providing new clues on the molecular and cellular mechanisms exploited to develop acquired resistance to EGFR-TKIs. One can also envisage that, complementing the advances made by STORM in clinical studies (e.g., [209]), methods with resolutions comparable to the size of the receptor, such as FLImP [85], might eventually be used in the clinic, for example, in assay groups of normal and cancerous cells (e.g., isolated from less invasive endobronchial ultrasound scan and biopsy (EBUS) [222] samples), from which the architecture of EGFR oligomers formed by the combination of wild-type and mutant receptors could be determined at the time of diagnosis. In addition, changes in oligomer architecture could then be potentially followed post-treatment with TKIs. If there was divergence between the changes in structure induced by TKIs in different EGFR oligomer cohorts, one can in principle analyse post-hoc the predictive potential of such oligomers’ structures. Indeed, recent work has highlighted the importance of structure–function relationships in predicting the response to TKIs with higher sensitivity than exon-based groups [127], so the future is almost here.
